# HISTORICAL REDLINING AND COGNITIVE FUNCTIONING AMONG US OLDER ADULTS

**DOI:** 10.1093/geroni/igad104.1929

**Published:** 2023-12-21

**Authors:** Calley Fisk, Jennifer Ailshire, Katrina Walsemann

**Affiliations:** University of Southern California, Los Angeles, California, United States; University of Southern California, Los Angeles, California, United States; University of Maryland, College Park, College Park, Maryland, United States

## Abstract

Historical redlining disproportionately harmed Black and disadvantaged communities by designating them as less desirable for mortgage lending. Residence in neighborhoods historically designated as less desirable is associated with worse health outcomes, though little research exists on older adults. Redlined neighborhoods often have characteristics that increase risk for poor cognitive function among older adults (e.g., greater air pollution and less greenspace). We examine differences in cognitive function trajectories by redlining status over 18 years (1998-2016) using data on 5,527 respondents ages 50 and older from the nationally representative Health and Retirement Study that have been linked to National Community Reinvestment Coalition Historic Redlining Scores Data. Results from multilevel linear regression models - adjusted for individual sociodemographic characteristics - show that baseline level of cognitive function is lower in neighborhoods historically designated as “Declining” (b=-0.61, p<.001) or “Hazardous” (b=-0.95, p<.001) compared with “Best or Desirable”. We do not find any association with cognitive change over time. Baseline differences were explained by educational attainment and income for residents of “Declining” neighborhoods, and tract-level affluence and disadvantage for residents of “Hazardous” neighborhoods. Race-stratified analyses showed that the impact of historical redlining on cognitive function was generally similar for Black and White adults, though we found no association between historical redlining and cognitive function for Black residents of “Declining” neighborhoods. Our findings suggest that redlining, as both an historical practice and process affecting the socioeconomic trajectories of individuals and neighborhoods, can shape cognitive health in older adults.

